# Flow Sorting, Whole Genome Amplification and Next-Generation Sequencing as Combined Tools to Study Heterogeneous Acute Lymphoblastic Leukemia

**DOI:** 10.3390/diagnostics13213306

**Published:** 2023-10-25

**Authors:** Rabiah Fardoos, Claus Christensen, Nina Friesgaard Øbro, Ulrik Malthe Overgaard, Bodil Als-Nielsen, Hans Ole Madsen, Hanne Vibeke Marquart

**Affiliations:** 1 Department of Clinical Immunology, Copenhagen University Hospital Rigshospitalet, DK-2100 Copenhagen, Denmark; 2 Department of Hematology, The University Hospital Rigshospitalet, DK-2100 Copenhagen, Denmark; 3 Department of Pediatric and Adolescent Medicine, Copenhagen University Hospital Rigshospitalet, DK-2100 Copenhagen, Denmark; 4 Faculty of Health and Medical Sciences, Institute of Clinical Medicine, University of Copenhagen, DK-2100 Copenhagen, Denmark

**Keywords:** B-cell precursor acute lymphoblastic leukemia, next-generation sequencing, cell sorting, whole genome amplification, immunoglobulin (IG)/T-cell receptor (TR) gene rearrangement

## Abstract

Next-generation sequencing (NGS) methods have been introduced for immunoglobulin (IG)/T-cell receptor (TR) gene rearrangement analysis in acute lymphoblastic leukemia (ALL) and lymphoma (LBL). These methods likely constitute faster and more sensitive approaches to analyze heterogenous cases of ALL/LBL, yet it is not known whether gene rearrangements constituting low percentages of the total sequence reads represent minor subpopulations of malignant cells or background IG/TR gene rearrangements in normal B-and T-cells. In a comparison of eight cases of B-cell precursor ALL (BCP-ALL) using both the EuroClonality NGS method and the IdentiClone multiplex-PCR/gene-scanning method, the NGS method identified between 29% and 139% more markers than the gene-scanning method, depending on whether the NGS data analysis used a threshold of 5% or 1%, respectively. As an alternative to using low thresholds, we show that IG/TR gene rearrangements in subpopulations of cancer cells can be discriminated from background IG/TR gene rearrangements in normal B-and T-cells through a combination of flow cytometry cell sorting and multiple displacement amplification (MDA)-based whole genome amplification (WGA) prior to the NGS. Using this approach to investigate the clonal evolution in a BCP-ALL patient with double relapse, clonal TR rearrangements were found in sorted leukemic cells at the time of second relapse that could be identified at the time of diagnosis, below 1% of the total sequence reads. These data emphasize that caution should be exerted when interpreting rare sequences in NGS experiments and show the advantage of employing the flow sorting of malignant cell populations in NGS clonality assessments.

## 1. Introduction

Acute lymphoblastic leukemia (ALL) is the most common cancer in childhood, with the highest incidence found between ages two and five [1]. V(D)J recombination has already occurred in the majority of malignant B- and T-cells, and clonally rearranged IG/TR genes are identifiable in 95% of ALL patients [2]. Correctly identifying clonal IG/TR rearranged genes in ALL patients allows for highly sensitive and specific PCR-based monitoring of the level of minimal residual disease during the course of treatment [3]. However, heterogeneity is a common phenomenon in BCP-ALL [4,5], raising concerns that identified IG/TR rearrangements are not uniformly present in all leukemic blasts at the time of diagnosis. The preservation of the IG/TR gene rearrangements is found in only 70% of BCP-ALL and 90% of T-ALL patients at the time of relapse [6,7], either because of continuing V(D)J recombinase activity in the malignant cells, or due to the limited sensitivity of the available methods for identifying IG/TR genes that cause minor subpopulations to be overlooked at the time of diagnosis. Of note, the metastasis-like dissemination of ALL cells to the central nervous system is a common cause of relapse in ALL [8], and in some patients, the IG gene rearrangements that dominantly present in the ALL cells in the CNS are found only at very low levels in the bone marrow at the time of diagnosis [9]. Improving the sensitivity of the IG/TR gene rearrangement analysis at the time of diagnosis could advance the detection of minimal residual disease over the course of treatment and improve the prediction of relapse in patients with heterogenous leukemia.

The EuroClonality/BIOMED-2 protocol is a multiplex PCR-based method that is widely used to identify clonal IG/TR rearrangements. The system is commercially available as IdentiClone assays (Invivoscribe), which were used in this study. The multiplex PCR reactions generate amplicons of 150–400 bp, which are subsequently investigated via fragment length (GeneScan) analysis or heteroduplex analysis, followed by direct sequencing or, in the case of bi- and oligoclonality, a combination of cloning and sequencing [10]. Recently, next-generation sequencing (NGS)-based methods have been developed and applied for the deep sequencing of IG and TR gene rearrangements in order to follow the normal B- or T-cell repertoire [11,12,13], to follow the clonal evolution of lymphoid malignancies using deep sequencing [14,15,16] or for monitoring minimal residual disease (MRD) [17,18,19,20]. The EuroClonality-NGS consortium has reported on the development of a method to comprehensively detect clonal IG/TR rearrangements [21,22], comparable in scope to the existing EuroClonality-BIOMED2 protocol. Here, we compared the EuroClonality-NGS and IdentiClone multiplex-PCR/GeneScan methods on a series of clonal BCP-ALL patient samples, and show that the NGS method is more sensitive than the multiplex-PCR/gene-scanning method. Furthermore, we show that whole genome amplification (WGA) is a feasible approach to identify clonal IG/TR rearrangements in samples with low cell numbers, and that flow cytometry cell sorting combined with WGA-NGS allows for a more detailed assessment of heterogenous leukemia.

## 2. Materials and Methods

### 2.1. Subjects

Eight BCP-ALL, patients treated and monitored in Denmark based on the standardized ALLTogether protocol, were retrospectively investigated in this study. In addition, one BCP-ALL patient had been diagnosed and treated according to the Nordic Society of Pediatric Hematology and Oncology (NOPHO) ALL2008 protocol until second relapse, after which the patient was diagnosed and treated according to the ALLTogether protocol [23]. Patient-specific clonal Ig/TR gene rearrangements were investigated according to the BIOMED-2 guidelines [3,10] and the leukemia-associated immunophenotypes were analyzed through flow cytometry using protocol-defined 6-color MRD panels, according to the NOPHO ALL2008 guidelines, [24] or 8-color MRD panels, according to the ALLTogether guidelines [25].

### 2.2. Sample Preparation for Clonality Detection via Next-Generation Sequencing

The EuroClonality-NGS assay, used for marker identification, is a two-step PCR protocol with eight primer sets (IGH-VJ, IGH-DJ, IGK-VJ-Kde, intron-Kde, TRB-VJ, TRB-DJ, TRG, TRD), as described in Brüggemann et al., 2019 [21]. In brief, sequencing libraries were prepared using a final reaction volume of 50 µL with 100 ng of diagnostic DNA and two types of spiked-in quality controls (QCs), comprising 10 ng of a poly-target quality control (cPT-QC) and 40 cell equivalents of DNA from nine human B- and T-cell lines, serving as an in-tube quality/quantification control (cIT-QC) [22]. After the 1st PCR, gel electrophoresis was performed to check for the successful amplification of all targets. For TRB, gel extraction of the specific PCR products was performed prior to the 2nd PCR. All first-round PCR products, except for the TRB PCR products, were diluted 50× unless the amplicons were very weak. The TRB PCR products and PCR products with weak amplicons were used undiluted. The primers for the 2nd PCR contained sequencing adaptors and sequencing indexes (barcodes). A unique combination of forward and reverse indexes was used for each library. Three µL of the undiluted TRB PCR products and 1 μL of 50×-diluted IGH, IGK, TRG and TRD PCR products were amplified in the 2nd PCR. After the PCR reactions were completed, the individual PCR reactions were pooled at similar ratios, and the PCR amplicons were purified using an Agencourt AMPure XP kit (Beckman Coulter, Brae, CA, USA). Finally, the sub pools were pooled equimolarly into one final pool (quantified with Qubit 3.0, Thermo Fisher Scientific, Waltham, MA, USA). Subsequently, paired-end sequencing (2 × 250 bp) was performed on an Illumina MiSeq (Illumina, San Diego, CA, USA), with a final concentration of 7 pM per library, aiming for at least 2000 reads per sample. To avoid low-complexity issues, a 10% PhiX control was added to each sequencing run. The sequence data sets were analyzed using ARResT/Interrogate (version 1.21.339) [26]. The identified IG/TR sequences were defined as index sequences if their abundance after cIT-QC normalization exceeded 5% or 1%.

### 2.3. IdentiClone Clonality Detection Based on EuroClonality BIOMED-2

The EuroClonality/BIOMED-2 approach to IG/TR gene rearrangement analysis was carried out as described [10] using IdentiClone Clonality Kits (InVivoScribe Technologies, San Diego, CA, USA). In case of clonal gene rearrangements, the PCR products were purified from 96-well plates using the Qiaquick PCR purification kit (Qiagen, Hilden, Germany), and were sequenced directly using biotinylated sequencing primers. The sequencing reactions were purified using streptavidin beads prior to loading onto an ABI 3130XL genetic analyzer. In case of bi-and oligoclonality, specific PCR products were cloned into TOPO-TA vectors and, following transformation into competent bacteria, the products were selected on agar containing tetracycline, X-Gal and IPTG. A selection of positive colonies was tested via PCR for the correct PCR-insert size. Sequencing was performed using biotinylated TOPO vector-specific T7 or M13 primers.

### 2.4. Cell Sorting via Flowcytometry

Cell populations were isolated from cryopreserved cells from diagnosis and relapse through flow-sorting on a FACSAria (BD) equipped with FACS Diva 8 software, as previously described [4], using 8-color FCM MRD panels. The sort gates were defined on the basis of the heterogeneity of the patient-specific leukemia-associated immunophenotype (LAIP) found in the blast populations in the routine flow diagnostic analysis. Cell sorting was performed at ‘high-speed’ with a 100 μm nozzle, a sheath pressure of 20 PSI, and in a 4-way-purity mode. The maximum possible number of cells was sorted into Falcon tubes containing 200 µL of FACSflow. When possible, in terms of the number of cells, a part of the sorted populations was reanalyzed, showing a sorting purity of at least 98%. Cell pellets of the sorted cell subpopulations were frozen at −80 °C.

### 2.5. Whole Genome Amplification and DNA Purification

The genomic DNA was amplified using a REPLI g Midi Kit (Qiagen, Germany) as described by the manufacturer. DNA from the diagnostic samples, sorted cell populations and WGA was purified using a Maxwell 16 LEV Blood DNA Kit on a Maxwell 16 purification robot (Promega, Madison, WI, USA). The DNA yield from the WGA ranged from 15 to 40 µg, depending on the input of genomic DNA. The purity of the amplified genomic DNA copies from the WGA reactions was 50–80%, with the rest being nonsense DNA, as estimated in a genomic single-copy gene RQ-PCR assay.

## 3. Results

### 3.1. Comparison between EuroClonality NGS and GeneScan for Clonality Assessment

**Figure 1:**
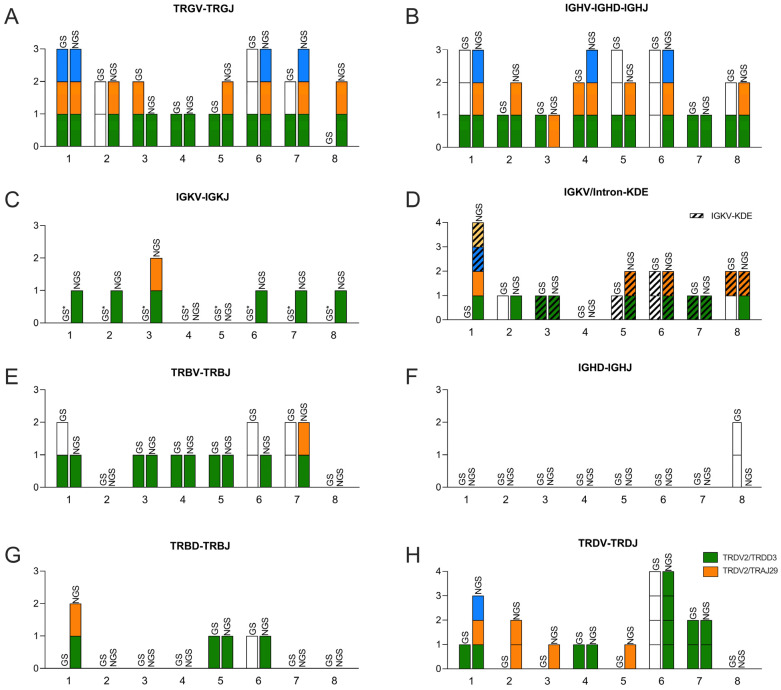
Number of clonotypes present in the mononuclear cell fraction of bone marrow samples at the time of diagnosis in leukemia patients (*n* = 8). Clonotypes were identified with the BIOMED-2/IdentiClone Clonality Kit GeneScan (GC) or EuroClonality NGS (EC). The number of identified clonotypes is presented on the y-axis and individual columns are divided into colored rectangles, each representing a particular clonotype. Within each figure element, clonotypes found in the same sample with both techniques are presented with the same color. No color indicates that a clonotype was identified with GeneScan, but not sequenced. (**A**): Complete TRG recombinations involving VDJ elements. (**B**): Complete IGH recombinations involving VDJ elements. (**C**): IGK recombinations involving V-J elements. (**D**): IGK recombinations involving V/Intron-Kde elements. (**E**): Complete TRB recombinations involving VJ elements. (**F**): Incomplete IGH recombinations involving DJ elements. (**G**): Incomplete TRB recombinations involving DJ elements. (**H**): Incomplete TRD recombinations involving VJ elements. GC not able to detect TRDV2/TRAJ29 (red). *, Polyclonal.

Initially, we investigated the performance of the EuroClonality NGS method with respect to IG/TR target identification in eight clinical samples, that all showed two or more peaks in one or more markers using the IdentiClone Clonality Kits GeneScan protocol. These samples represented more challenging clinical cases compared to the common homogenous BCP-ALL cases (Table 1). When several peaks were identified for a marker using the GeneScan method, the identified fragments were only subjected to cloning and sequencing if other clonal markers could not be identified. In comparison, the EuroClonality NGS method used the Arrest/Interrogate software (version 1.99.8) for data analysis and had the advantage of immediately providing the sequences. Table 1 summarizes the number of clonotypes found using the two methods. Figure 1 illustrates when the same clonotype was identified using both methods.

When operated with the standard 5% threshold, the EuroClonality NGS showed superior sensitivity compared to GeneScan in the case of the TRG VJ and IGH markers, as well as IGK (Figure 1A–C). In the case of TRG VJ, the EuroClonality NGS method identified one or two additional gene rearrangements compared to the GeneScan method in three out of eight samples (Table 1), and those targets that were identified via sequencing using the GeneScan method were all found using the NGS method (Figure 1A). In the case of IGH, the EuroClonality NGS method identified additional gene rearrangements compared to the GeneScan method in two samples (samples 2 and 4) (Figure 1B). Also, in the case of IGK, the NGS method identified more clonotypes than the GeneScan method (Figure 1C). However, the routine GeneScan laboratory set-up only included the detection of incomplete V-intron/Kde rearrangements. In contrast, the NGS method made use of two primer sets, A and B, of which A includes all IgK gene rearrangements. For this reason, a direct comparison was not reasonable.

Clonal TRB gene rearrangements were found in six out of eight samples using the GeneScan method, but the detection of TRB recombination events represented a particular challenge for the NGS method. In the EuroClonality protocol, it was necessary to include the gel extraction of the specific TRB products after the first PCR before proceeding to the second PCR. Only following this approach, the EuroClonality NGS method performed at the same level as GeneScan, identifying the same number of clonotypes. A notable exception was samples 1 and 6, in which the GeneScan method identified one potential clonotype more than the NGS method (Figure 1E).

As expected, incomplete DJ gene rearrangements in IGH and TRB were less common in the patient samples. Hence, GeneScan identified incomplete IGH DJ rearrangements in one sample (sample 8), incomplete TRB DJ rearrangements in two samples (samples 5 and 6) and incomplete TRD VD rearrangements in four out of eight samples. The EuroClonality NGS was not able to identify any clonotypes in the case of IGH DJ (Figure 1F), but did find additional TRB DJ clonotypes compared to GeneScan (two clonotypes in sample 1, Figure 1G). With respect to TRD, the EuroClonality NGS also identified additional clonotypes compared to GeneScan in four out of eight samples. The reason for the identification of these additional clonotypes was due to the EuroClonality NGS detecting TRDV2/TRAJ29 clonotypes, because this setup includes primers binding TRAJ29, which is not part of the GeneScan set-up. Also of interest was sample 6, which represented a striking case of oligoclonality in TRD, with at least four different TRD V2-D3 fusions identified with both methods (Figure 1H).

Overall, EuroClonality NGS identified 17 clonotypes more than GeneScan, representing 29% more identified targets across all samples and markers (Table 1). In NGS experiments, a major determining factor for the number of clonotypes identified is the so-called threshold. This is a prespecified percentage of the total reads that is used as a cut-off to discriminate clonotypes that are likely associated with malignant cells from the normal background of gene rearrangements present in normal B- and T-cells within each sample. In Table 1 and Figure 1, the threshold was set to 5%. However, merely lowering the threshold to 1% resulted in an increase in the number of potential clonal rearrangements from 76 to 141 across all samples and markers. As expected, large inter-sample differences were observed when, for example, lowering the threshold from 5% to 1%, which resulted in 24 additional clonotypes in the case of sample 6, but only one additional TRG VJ clonotype in the case of sample 2 (Supplementary Table S1).

### 3.2. Clonality Assessment by Using WGA for Low-Input DNA Samples

**Figure 2:**
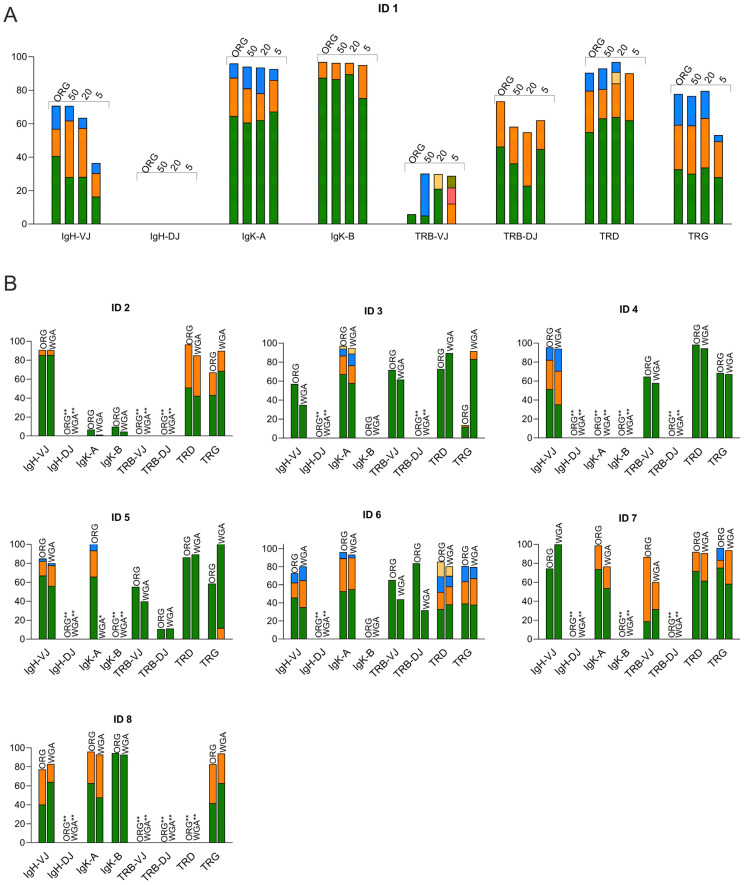
NGS-based IG/TR clonality assessment using genomic DNA after whole genome amplification (WGA). (**A**): Clonotypes in sample 2 identified using original, non-amplified DNA (ORG) and WGA-DNA generated from 50 ng, 20 ng and 5 ng, respectively. Clonotypes are color-coded within each separate marker and the quantity of the clonotype is depicted as the percentage of total reads. (**B**): Direct comparison between 50 ng of input DNA and 50 ng of WGA DNA (*n* = 9). *, not assessed; **, no clonal product.

In the above analysis, 50 ng of genomic DNA was used as the input material for each of the reactions subjected to NGS, requiring a total of 400 ng to test all available markers. In experiments aiming at IG/TR target identification in subpopulations isolated from heterogenous leukemic samples, this requirement could pose a limitation. Therefore, we investigated whether WGA could be a viable approach to increase the DNA prior to NGS. Experiments were made using 5, 20 or 50 ng of gDNA from sample 2 as the starting material for multiple displacement amplification (MDA)-based WGA (REPLIg, Qiagen, Germany), followed by the addition of 50 ng of amplified DNA in each reaction subjected to NGS. The results showed that WGA could reproduce the pattern of the original, non-amplified sample, except for the marker TRB DJ. The reproduction of clonotypes was possible, even when using 5 ng of gDNA as the starting material for WGA, but in general, 50 ng of gDNA was needed to avoid the emergence of additional clonotypes that were not readily seen in the non-amplified sample (Figure 2A). Consequently, for the remaining seven samples presented in Figure 1, we used 50 ng of DNA for WGA, followed by the addition of 50 ng of amplified DNA in each of the NGS reactions. Direct comparisons led to similar findings as for sample 2. Whenever particular gene rearrangements appeared to be clonally present, the clonotypes identified via the NGS of non-amplified DNA could consistently be reproduced through the NGS of the corresponding WGA-DNA (Figure 2B). Overall, this analysis showed that WGA could be used to generate higher amounts of DNA for the NGS-based analysis of IG/TR gene rearrangements, but caution was warranted when using less than 50 ng of DNA for WGA.

### 3.3. Analysis of Relapse in a BCP-ALL Patient Using Flow Sorting, WGA and NGS

**Figure 3:**
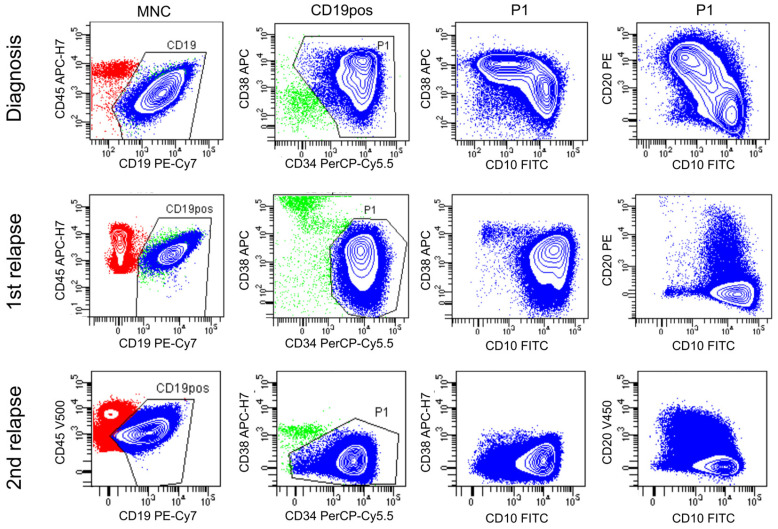
Flow cytometric immunophenotyping showing the heterogenous expression of surface markers in a BCP-ALL patient. The surface expression of CD45, CD19, CD38, CD34, CD20 and CD10 are depicted at the time of diagnosis, first relapse and second relapse. After initial gating for singlets and mononuclear cells (MNCs) in FSC/SSC plots, CD19-positive cells were gated. Malignant blasts (P1) were further defined in CD34/CD38 plots. At diagnosis, the malignant blast cells showed unimodal expression of CD34 and bimodal expression of CD38, CD20 and CD10. At relapse time points, malignant blasts were predominantly CD34pos, CD38dim, CD10pos and CD20neg, with less than 10 percent of the cells showing CD20dim and CD10neg/dim expression. Blue, leukemic blasts; green, non-malignant B-lineage cells; red, MNCs. At diagnosis and first relapse, cells were analyzed using a NOPHOALL2008 protocol antibody combination, while at second relapse, an antibody combination from the A2G protocol was used.

To investigate the combined power of cell sorting, WGA and NGS in the examination of heterogenous BCP-ALL, we selected a case of heterogenous, recurring BCP-ALL. This patient was initially diagnosed with BCP-ALL, and later experienced two relapses; the first was after nearly two years and the second was five years after the initial diagnosis. According to the initial flowcytometric analysis, the blasts were homogenously positive for CD19 and CD34, but were heterogenous with respect to several other surface markers, including CD38, CD10 and CD20. At the time of the first relapse, the recurred malignant clones were still CD34pos and CD19pos, as well as, predominantly, CD38neg/dim, CD10pos and CD20neg. This marker profile was again observed on the blasts reemerging at the time of the second relapse, except with regard to CD38, which was entirely negative. At both timepoints, approximately 10% of the malignant blasts showed dim CD10 and weak CD20 expression (Figure 3). Collectively, the flowcytometric analysis indicated that the CD10pos/CD38neg/CD20neg leukemic blasts, seen at diagnosis, were the dominant cause of both relapses, yet a minor fraction of the cells were CD10dim and CD20dim.

**Figure 4:**
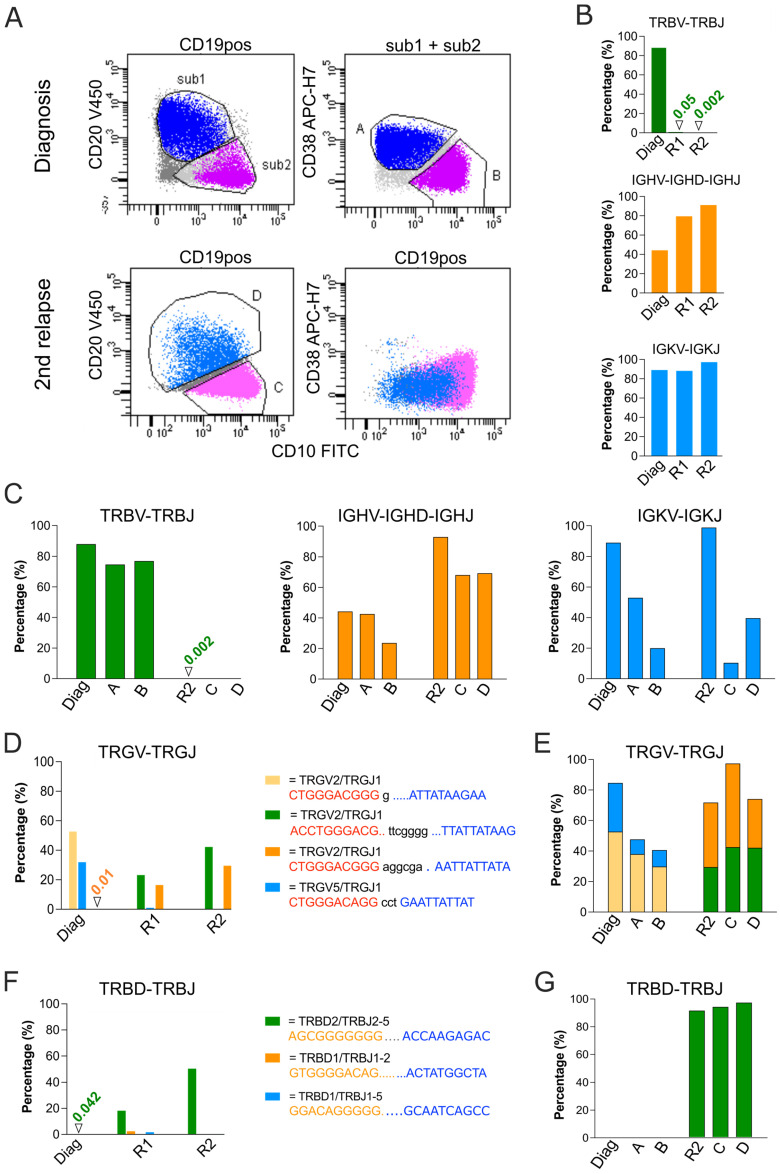
NGS-based assessment of IG/TR gene rearrangements in flow-sorted subpopulations of leukemic blasts from a BCP-ALL patient at the time of diagnosis and relapses. (**A**): Flow cytometry dot plots showing the surface expression of CD38, CD10 and CD20 in leukemic blasts at the time of diagnosis (top) and second relapse (bottom). Blasts were sorted via flow cytometry, corresponding to the following expression profiles: CD38pos/CD10neg, dim (A), CD10pos/CD38 dim (B), CD10pos/CD20neg (C) and CD20dim/CD10neg (D). (**B**): DNA isolated at diagnosis, and at first and second relapse, were subjected to NGS-based analysis of gene rearrangements in TRB (TRBV-TRBJ), IGH (IGHV-IGHD-IGHJ), and IGK (IGKV-IGKJ and IGKV/Intron-KDE). The columns indicate the percentage of total reads of each clonotype. (**C**): NGS-based analysis of the same markers as in A, comparing clonotypes found in crude bone marrow samples at the time of diagnosis and second relapse with those found in sorted leukemic populations from the same samples. (**D**,**F**): DNA isolated at diagnosis, first and second relapse were subjected to NGS-based analysis of gene rearrangements in TRG (TRGV-TRGJ) and TRD (TRDV-TRDJ). (**E**,**G**): NGS-based analysis of the same markers as in (**D**,**F**), comparing clonotypes found in crude bone marrow samples at the time of diagnosis and second relapse with those found in sorted leukemic populations from the same samples. In (**B**–**G**), columns show the abundance of clonotypes above the 5% threshold, with the y-axis depicting the percentage of total reads. The sequences of the clonotypes are shown in (**D**,**F**), with the junction sequence in lowercase and flanking sequences in capitals. Red, V-element; blue, J-element, yellow, D-element. Diag, diagnosis; R1, first relapse; R2, second relapse.

The GeneScan analysis was performed at the time of diagnosis, and following the identification of clonal rearrangements in TRB and IGH, sequencing identified the TRB recombination as VB20-1/JB2.3 and the IGH as VH3-71/DH6/JH6. These markers were chosen as PCR markers to be used for later MRD analysis. Upon the first relapse, the TRB VB20-1/JB2.3 was lost, leaving the IGH marker as the only useful MRD-PCR marker. Yet, as shown in the flowcytometric marker profile in Figure 3, the leukemia was heterogenous at the time of diagnosis and at both relapses, raising questions as to whether the IGH marker was present in all subpopulations and whether other rearrangements had occurred in some of the subpopulations upon relapse. To address these questions, leukemic cells were sorted at the time of diagnosis and second relapse according to the CD38, CD10 and CD20 expression status. At diagnosis, cells were sorted into CD38pos CD10neg/dim cells (subpopulation A), and CD38neg CD10pos cells (subpopulation B), whereas at second relapse, cells were sorted into CD20neg CD10pos (subpopulation C), and CD20dim CD10neg (subpopulation D) (Figure 4A). Genomic DNA was subjected to WGA and analyzed with NGS alongside the unsorted, non-amplified diagnostic and second relapse samples. NGS sequencing identified the clonal TRB recombination event as V20-1/J2.3 and confirmed the almost complete loss of this marker at both relapse time points, meanwhile confirming the persistence of the IGH (V3-71/D6/J6) marker in both relapse samples. Also, NGS identified IgK V7-3/J2 and Intron/Kde clonotypes in the leukemic blasts at diagnosis and at relapse timepoints (Figure 4B). Whilst the IGK V/J and IGK intron/Kde markers were present in all of the sorted populations from both the diagnostic and relapse samples, the TRB marker was detected in both subpopulations in the diagnostic sample, but in none of the subpopulations in the second relapse sample (Figure 4C).

With regard to the TRG marker, NGS identified two different TRG-VG2/JG1 recombination events at the two relapse time points (Figure 4D). Both existed within WGA-DNA from sorted cells representing CD20neg CD10pos, and CD20dim CD10neg leukemic subpopulations. This confirms the malignant origin of these clonotypes as well as pointing to a common clonal origin of these subpopulations despite differences in the surface markers (Figure 4E). Interestingly, NGS failed to detect both TRG-V2/J1 clonotypes at the time of diagnosis above the 5% threshold. Whilst the TRG-VG2/JG1 (0/6/−1) clonotype was detected at a level of 0.01%, the TRG VG2/JG1 (−2/7/−3) was below the detection limit (Figure 4D). A similar situation existed for the TRB incomplete rearrangement, which was identified as TRB- D2/J2.5 in both relapse samples, irrespective of whether they were sorted according to CD20/CD10 status or not. Again, this clonotype was found at the time of diagnosis at a level of 0.04% of the total sequencing reads (Figure 4F,G). Of note, both the TRG and the TRB-DJ were observed using the GeneScan method in the relapse samples, but this method failed to detect these gene rearrangements in the diagnostic sample.

Collectively, the NGS data supported the clonal origin of the blasts that gave rise to relapse. It is noteworthy that NGS was able to detect the pre-existence of these cells as minor subclones at the time of diagnosis, judging from the detection of markers well below the 5% threshold, which is commonly used to distinguish malignant cells from the background of normal B- and T-cells.

## 4. Discussion

Improving the ability to the detect minor subpopulations of malignant cells is instrumental to the achievement of the greater overall survival of patients with heterogenous leukemia, yet it requires more sensitive methods for comprehensive marker identification in malignant cells at the time of diagnosis. Until now, a preferred technique for IG/TR marker identification in ALL patients has been the EuroClonality/BIOMED-2 protocol. It consists of a series of multiplex PCRs targeting a selection of IG and TR genes, followed by GeneScan analysis to identify the clonally rearranged genes. This is followed by Sanger sequencing, or in the case of bi/oligoclonality, a combination of cloning and sequencing using gene-specific primers. As such, the multiplex PCR/GeneScan method is a relatively low-throughput and complicated technique. The EuroClonality NGS method provides the sequences of the identified IG/TR gene rearrangements as part of the workflow with no need for cloning. This proves to be particularly important when dealing with heterogenous leukemia with multiple subclones harboring different IG/TR gene rearrangements. Furthermore, the EuroClonality-NGS encompasses a larger set of primers covering all types of complete and incomplete TRD recombinations, including VD-JA29, which is estimated to be present in 20% of BCP-ALL cases [27]. A largescale validation of the EuroClonality-NGS in the context of ALL reported that this NGS method identified, on average, 4% more markers per patient than BIOMED-2. Here, TRDV2-JA29 recombinations were detected in 24% of patients [21]. In a technical feasibility study focusing on frozen and formalin-fixed paraffin-embedded samples from 10 B-cell lymphoma patients, the NGS method identified 22% more clonal rearrangements than BIOMED-2/GeneScan [28]. In our study, based on eight BCP-ALL samples and including eight markers, the EuroClonality-NGS method identified 17 more clonotypes than the BIOMED-2/GeneScan protocol, representing 29% more identified targets. Partly, this was due to the ability of EuroClonality NGS to detect TRDV2-JA29 rearrangements, which were observed in four of eight patients. Furthermore, the IGK-VJ was not included in our use of the GeneScan method, causing fewer clonal IGK rearrangements to be found.

One of the key parameters affecting the number of clonotypes identified with NGS is the threshold used when interpreting the results. In the present study, lowering the threshold from 5% to 1% resulted in an increase in the number of potential clonal rearrangements from 76 to 141 across all samples and markers, but this substantial overall increase encompassed large inter-patient variation. Ideally, the threshold should properly distinguish IG/TR rearrangements from the background of infiltrating normal B- and T-cells, which may be present in patient samples to different extents. The available NGS studies have consistently used thresholds of 5% or higher, but it is difficult to specify the correct threshold that allows for the unambiguous assignment of clonotypes to ALL [29,30]. In support of a 5% threshold, a study of lymph nodes and tonsil samples dominated by reactive lymphoproliferations, the most abundant clonotype, was shown to have a frequency of <5% of the reads [31]. However, a recent study indicated that many IGH gene rearrangements, with shared stem D_H_-N additions-J_H,_ are detectable in BCP-ALL samples below the 5% threshold. Allegedly, these sequences represent gene rearrangements that underwent continued D_H_/V_H_-DJ_H_ recombination and V_H_ replacement, [32]. Hence, whilst a high threshold might guarantee specificity in the overall assignment of clonotypes to ALL, it comes with a risk of overlooking a plethora of less abundant, potentially therapy-resistant subclones that could give rise to relapse. Here, we propose the use of cell sorting in combination with NGS-based IG/TR gene rearrangement analysis that, in part, represents a way to circumvent the threshold issue. By taking advantage of aberrant surface markers on malignant cells, the flow-based separation of malignant cells will greatly reduce the presence of normal cells. In most of ALL cases, this may be a feasible approach, considering that more than 90% of both T-ALL and B-ALL cases have one or more markers distinguishing them from normal mature and precursor lymphocytes [24,33]. In the case of the relapsing leukemia reported here, the separation of malignant cells from background lymphocytes based on CD38, CD10 and CD20 expression allowed for the immediate assignment of IG/TR markers to malignant cells, both at diagnosis and at relapse. Importantly, some clonotypes, i.e., TRGV2/J1 (−0, 6, −1) and TRB-D2/J2.5, were found in the diagnostic sample below the 5% threshold, whereas other clonotypes were below the detection level in the diagnostic sample. This illustrates the problem of assigning clonotypes to background lymphocytes simply because they are represented by less than a pre-specified percentage of the total sequence reads. Rather than specifying such a threshold, we propose that cell sorting via flow cytometry is used to limit the analysis to cells with aberrant surface markers and to ensure that rare sequences are also considered. This will increase the likelihood of identifying the recombination events that are present in rare subclones, which otherwise escape notice and potentially give rise to relapse.

First and foremost, cell numbers restrict the ability to make a full NGS-based IG/TR gene rearrangement analysis of sorted leukemic subpopulations. The EuroClonality-NGS protocol works optimally with 50 ng of DNA input in each reaction, amounting to a minimum requirement of 400 ng of gDNA for a complete analysis [31]. In comparison, 600 ng is the expected amount of gDNA from 100,000 diploid cells. Such numbers may simply be unachievable for minor leukemic subpopulations within clinical samples, necessitating some form of DNA amplification prior to NGS. WGA has been used widely in cancer diagnostics, e.g., to amplify gDNA from very limited numbers of circulating tumor cells, and the use of WGA-DNA for downstream NGS applications has been verified [34]. Here, we show that multiple displacement amplification using the REPLIg WGA protocol was able to reproduce the clonotypes in BCP-ALL samples. However, reducing the amount of input DNA for WGA below 50 ng led to a skewed proportion of clonotypes and the emergence of clonotypes that were not seen in the non-amplified samples. Issues related to non-uniform coverage due to regional differences in GC content resulting in allelic drop-out or imbalance, the generation of chimeric fragments and the introduction of false nucleotide variants due to DNA polymerase infidelity are well known phenomena associated with WGA [35]. These problems associate with different WGA protocols to different extents, but MDA-based WGA approaches, in particular the REPLIg WGA, have been shown to outperform other WGA methods with respect to DNA yield, amplicon size and amplification uniformity [36,37,38]. Still, as it relies on the binding of random hexamers for amplification, the REPLIg WGA is sensitive to differential primer binding efficiency. Given that REPLIg WGA, like other MDA methods, involves progressive hyper-branching, initial primer binding non-uniformity will impact the final result [35]. Allegedly, the observed failure of WGA to correctly represent the proportions of clonotypes could be related to differences in GC content between individual V, D and J elements, leading to the non-uniform binding of primers and, henceforth, non-uniform coverage.

## 5. Conclusions

In conclusion, we have shown that the sorting of malignant subpopulations, followed by WGA and NGS-based IG/TR gene rearrangement analysis, can be used to verify that IG/TR clonotypes are associated with malignant cells. This approach can verify markers of malignancy, even when they are present as only a few reads in NGS runs, without the use of a predetermined threshold to discriminate normal lymphocytes and leukemic blasts. Combining NGS with flow cytometry cell sorting at the time of diagnosis would be too laborious to apply to all patients. However, we envision that this strategy should be used primarily when conventional gene scanning and flow cytometry have identified a case of heterogenous leukemia with multiple subclones. In these complex cases, this approach could help to identify IG/TR markers in minor subclones to prevent their escape from detection and later return in the form of a relapse. In turn, this could lead to an improved overall survival of leukemia patients.

## Figures and Tables

**Table 1:** Number of clonotypes present in the mononuclear cell fraction of bone marrow samples at the time of diagnosis in leukemia patients (*n* = 8). The number of clonotypes was based on IdentiClone Clonality Kits GeneScan (GC), or EuroClonality NGS (NGS). P, polyclonal; PB, polyclonal background; O, oligoclonal.

	IGH VDJ		IGH DJ		IGK VJ + IGK V/Intron-Kde			
	GS	NGS	GS	NGS	GS	NGS	NGS	
ID 1	3	3	P	-	P	1	4	
ID 2	1	2	P	-	1	1	1	
ID 3	1	1	P	-	1	2	1	
ID 4	2	3	P	-	P	-	-	
ID 5	3	2 PB	P	-	1	-	2	
ID 6	3	3 PB	P	-	2	1	2	
ID 7	1	1	P	-	1	1	1	
ID 8	2	2	2	-	2	1	2	
	TRB VJ		TRB DJ		TRG VJ		TRD VJ	
	GS	NGS	GS	NGS	GS	NGS	GS	NGS
ID 1	2	1	P	2	3	3	1	3
ID 2	P	-	P	-	2	2	P	2
ID 3	1	1	P	-	2	1	P	1
ID 4	1	1	P	-	1	1	1	1
ID 5	1	1	1	1	1 PB	2	P	1
ID 6	2	1	1	1	3	3	4 O	4
ID 7	2	2	P	-	2	3	2	2
ID 8	P	-	P	-	P	2	P	P

## Data Availability

The NGS data presented in this study are available on request from the corresponding author. Patient data are not shared due to ethical restrictions.

## Supplementary Materials

The following supporting information can be downloaded at: https://www.mdpi.com/article/10.3390/diagnostics13213306/s1, Table S1: Number of clonal IG/TR gene rearrangements and its dependence on NGS threshold.
