# Supplementary material for: Flow Sorting, Whole Genome Amplification and Next-Generation Sequencing as Combined Tools to Study Heterogeneous Acute Lymphoblastic Leukemia
Source: Diagnostics (Basel). 2023 Oct 25;13(21):3306. doi: 10.3390/diagnostics13213306 (PMC10650172; doi:10.3390/diagnostics13213306)
Supplement: Supplementary file 1 [file diagnostics-13-03306-s001.zip › diagnostics-2659674-supplementary.pdf]

Supplementary Table S1 depicts the number of IG/TR rearrangements using a 5% and 1% threshold in ARResT/Interrogate. PB, polyclonal background; O, oligoclonal

	IGH VDJ		IGH DJ		IGK VJ + IGK V/ Kde		Intron Kde	
	5%	1%	5%	1%	5%(A)	1%(A)	5%(B)	1%(B)
ID 1	3	8	-	-	3	4	2	2
ID 2	2	2	-	-	1	1	1	1
ID 3	1	1	-	-	3	4	-	-
ID 4	3	3	-	-	-	-	-	-
ID 5	2 PB	11 PB	-	-	2	6	-	-
ID 6	3 PB	14 PB	-	-	3	3	-	-
ID 7	1	2	-	-	2	3	-	-
ID 8	2	5	-	-	2	4	1	2

	TRB VJ		TRB DJ		TRG VJ		TRD VJ	
	5%	1%	5%	1%	5%	1%	5%	1%
ID 1	1	1	2	2	3	7	3	3
ID 2	-	-	-	-	2	3	2	2
ID 3	1	1	-	-	1	2	1	1
ID 4	1	1	-	-	1	1	1	1
ID 5	1	1	1	1	2	2	1	1
ID 6	1	4	1	1	3	7	4 O	10 O
ID 7	2	2	-	-	3	4	2	3
ID 8	-	-	-	-	2	4	-	-
